# Plasmonic mid-infrared third harmonic generation in germanium nanoantennas

**DOI:** 10.1038/s41377-018-0108-8

**Published:** 2018-12-12

**Authors:** Marco P. Fischer, Aaron Riede, Kevin Gallacher, Jacopo Frigerio, Giovanni Pellegrini, Michele Ortolani, Douglas J. Paul, Giovanni Isella, Alfred Leitenstorfer, Paolo Biagioni, Daniele Brida

**Affiliations:** 1 Department of Physics and Center for Applied Photonics, University of Konstanz, 78457 Konstanz, Germany; 2 School of Engineering, University of Glasgow, Rankine Building, Oakfield Avenue, Glasgow, G12 8LT UK; 3 L-NESS, Dipartimento di Fisica del Politecnico di Milano, Via Anzani 42, 22100 Como, Italy; 4 Dipartimento di Fisica, Politecnico di Milano, Piazza Leonardo da Vinci 32, 20133 Milano, Italy; 5 Department of Physics, Sapienza University of Rome, 00185 Rome, Italy; 6 Physics and Materials Science Research Unit, University of Luxembourg, 162a avenue de la Faïencerie, L-1511 Luxembourg, Luxembourg

## Abstract

We demonstrate third harmonic generation in plasmonic antennas consisting of highly doped germanium grown on silicon substrates and designed to be resonant in the mid-infrared frequency range that is inaccessible with conventional nonlinear plasmonic materials. Owing to the near-field enhancement, the result is an ultrafast, subdiffraction, coherent light source with a wavelength tunable between 3 and 5 µm, and ideally overlapping with the fingerprint region of molecular vibrations. To observe the nonlinearity in this challenging spectral window, a high-power femtosecond laser system equipped with parametric frequency conversion in combination with an all-reflective confocal microscope setup is employed. We demonstrate spatially resolved maps of the linear scattering cross section and the nonlinear emission of single isolated antenna structures. A clear third-order power dependence as well as mid-infrared emission spectra prove the nonlinear nature of the light emission. Simulations support the observed resonance length of the double-rod antenna and demonstrate that the field enhancement inside the antenna material is responsible for the nonlinear frequency mixing.

## Nanoantennas: Concentrated infrared light source for molecular analysis

A nanoantenna resonant in the mid-infrared frequency range demonstrates promise for optical devices that analyze molecules. Mid-infrared frequency light can be used to detect molecular vibrations, enabling detailed analyses of individual molecules at the quantum level, which is useful for medical and environmental applications. Previous plasmonic nanoantennas developed to concentrate such light and drive nonlinear processes were not effective at Mid-infrared wavelengths. Now, Daniele Brida and co-workers have built a plasmonic nanoantenna using highly-doped germanium grown on silicon substrates, which acts as an ultrafast, tunable nonlinear light source when it is excited by a laser. Their nanoantenna is capable of third harmonic generation – the creation of a light wave with a tripled frequency (one third of the original wavelength) - allowing for enhanced interaction of light and molecules in the Mid-infrared.

## Introduction

Plasmonic nanoantennas^1,2^, i.e., resonant metallic structures with sub-optical-wavelength sizes, are one of the key components in advanced nano-optical applications. By directing and concentrating far-field electromagnetic radiation into subdiffraction-limited near-field volumes, plasmonic nanoantennas are ideal tools for accessing single quantum systems with light. Another benefit of the light concentration capabilities of resonant antennas is the ability to access nonlinear optical phenomena^3^ even with minute electromagnetic field amplitudes. This approach has been exploited successfully in the near-IR spectral range to generate second harmonic^2^, third harmonic^4^, or even higher order phenomena^3,5^. Alternatively, mid-IR plasmonic metamaterials have been employed to improve the coupling of far-field radiation with the nonlinearity of quantum wells^6^, dielectric nanostructures^7^, graphene^8^, or emerging mid-IR waveguides made of silicon nitride^9^ can be used for the nonlinear generation of mid-IR or terahertz photons. In the case of graphene, a high degree of 2D confinement and optical field manipulation can be reached in the mid-IR region^10^.

In recent years, highly doped semiconductors such as InAs^11^, InAsSb^12^, InP^13^, and Ge^14–16^ have been introduced as high-quality and tunable mid-IR plasmonic materials for integrated devices. The mid-IR frequency range is of notable interest for the chemical and biological identification of molecules for environmental, healthcare, and security sensing applications, since this range includes the so-called molecular fingerprint region^15,17–19^ with countless unique vibrational absorption lines. In this context, nonlinear plasmonics can be a sensitive and versatile tool for achieving a near-field tunable source that can directly interact with molecules at the nanoscale. Until now, however, intrinsic nonlinearities in plasmonic structures have not been demonstrated in the mid-IR spectral region since traditional materials are not effective at wavelengths longer than the typical near-IR and visible range^20^.

**Fig. 1:**
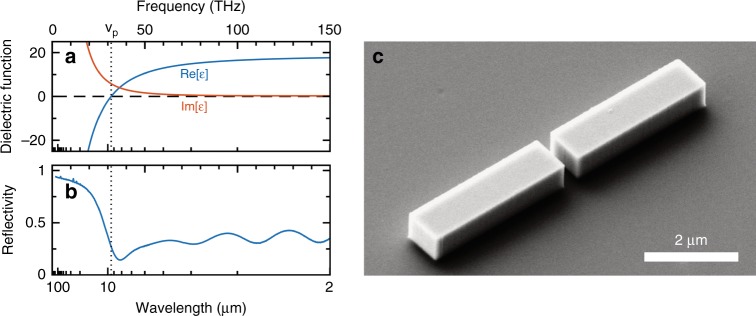
Plasmonic antennas from heavily doped germanium. **a** The complex dielectric function of a heavily doped germanium film extracted from **b**, the reflectivity spectrum. **c** A scanning electron micrograph of a single germanium double-rod antenna on a silicon substrate with an arm length of 3.5 µm, a thickness of 700 nm and a gap width of 300 nm

For several reasons, Ge is an ideal material for linear and nonlinear plasmonic applications in the mid-IR spectral range since, in recent years, extremely heavily doped materials with a tunable plasma frequency of up to 95 THz (3.1 µm wavelength) and high crystalline quality became available through epitaxial growth on silicon wafers^16^. The reflectivity and dielectric functions of the material employed in the reported experiments are depicted in Fig. 1a and b and correspond to a plasma frequency of ~9.7 µm with an active n-doping concentration of 2.5 × 10^19^/cm^−3^. In particular, the full CMOS compatibility of Ge-on-Si promises easy integration with the potential for inexpensive mass production. Moreover, the third-order nonlinear coefficient^21,22^ is comparable to that displayed by gold at visible and near-IR frequencies^23^. Since Ge is a nonpolar elementary semiconductor, the lack of optical phonon absorption reduces losses in the mid-IR region compared to III–V semiconductors. Finally, the plasma frequency can also be controlled dynamically by optical excitation of electron-hole pairs^24^.

In this work, we investigate the local generation of third harmonic radiation in the mid-IR region in plasmonic antennas with subwavelength dimensions. Our experiments enable several exciting perspective applications from nonlinear-enhanced sensing of molecular species^25^ to ultrafast near-field microscopy^26,27^ with unprecedented temporal and spatial imaging resolution for molecular-selective imaging in life sciences as well as the direct porting of a large variety of near-infrared nonlinear plasmonic applications^3^ in the mid-infrared region. Furthermore, the reported technique offers new ways to study the fundamental mechanisms of nonlinearity in condensed matter, especially in nanostructures^23,28^.

Nevertheless, the study of nonlinear plasmonics in the mid-IR wavelength range poses major challenges with respect to the near-IR and visible cases. Diffraction, which limits the excitation field in the focus, combines with the increased antenna interaction volume and leads to a complex, unfavorable wavelength scaling of the expected third harmonic generation (THG). In addition, the longer pulse durations at low frequencies further decrease the peak fields and thus the nonlinear emission. These difficulties require to be mitigated by scaling the laser system used for optical excitation. Under these conditions, Ge displays the additional advantage of having strong durability, while in metals, strong electromigration and diffusion prevent high-field excitation for nonlinear mid-IR studies^20^.

## Results

**Fig. 2:**
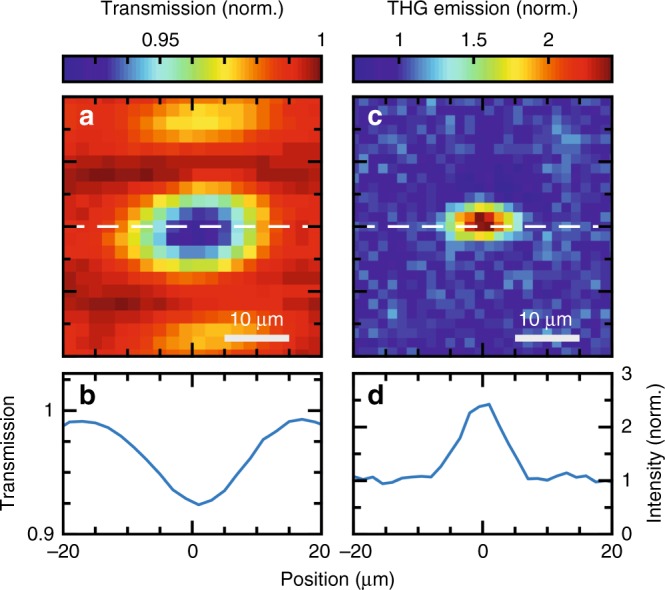
Linear and nonlinear confocal microscope images of a single antenna. **a** The spatially resolved transmission map of a heavily doped Ge double-rod antenna with an arm length of 4.5 µm at an excitation wavelength of 12 µm recorded by moving the antenna sample through the common objective focus. The darker regions around the antenna are diffraction artifacts of the reflective objectives complex point spread function. **b** Cut of the transmission map through the center of the antenna (along the white dashed line). **c** Spatially resolved third harmonic emission intensity from this antenna normalized to the substrate background emission. **d** Cut through the emission map

A high-power femtosecond laser system is employed to obtain narrowband, intense, mid-IR transients via difference frequency generation tunable over the wavelength range from 7 to 20 µm (for more details see methods section). At a pulse energy of up to 100 nJ, the mid-IR driving pulses feature a duration of 300 fs at a bandwidth of 1.5 THz. Single antenna structures (Fig. 1c) are excited in a dispersion-free Cassegrain-geometry reflective microscope setup with these multi-THz transients reaching peak electric fields of up to 5 MV/cm. By scanning the sample through the focal region in a transmission geometry, maps with high spatial resolution can be recorded. Figure 2a demonstrates the transmission map of a double-rod antenna with an arm length of 4.5 µm. The excitation wavelength is set to ~12 µm, corresponding to 25 THz. The extinction of this single resonant subwavelength structure exceeds 8% (Fig. 2b). Due to the subwavelength dimension of the antennas, the geometric width roughly represents the point spread function of the optical excitation. To discriminate between the fundamental and third harmonic radiation, crystalline filters are employed. This allows the strongly localized third harmonic emission from the plasmonic structure to be selected (Fig. 2c, d). The emission exceeds the substrate background by a factor of 2.5, which is substantial considering the small interaction volume of the driving beam with the subwavelength structure in comparison to the bulk silicon wafer interaction path. Both the excitation and the emitted third harmonic generated radiation are polarized along the antenna axis (see supplementary information Fig. S3).

**Fig. 3:**
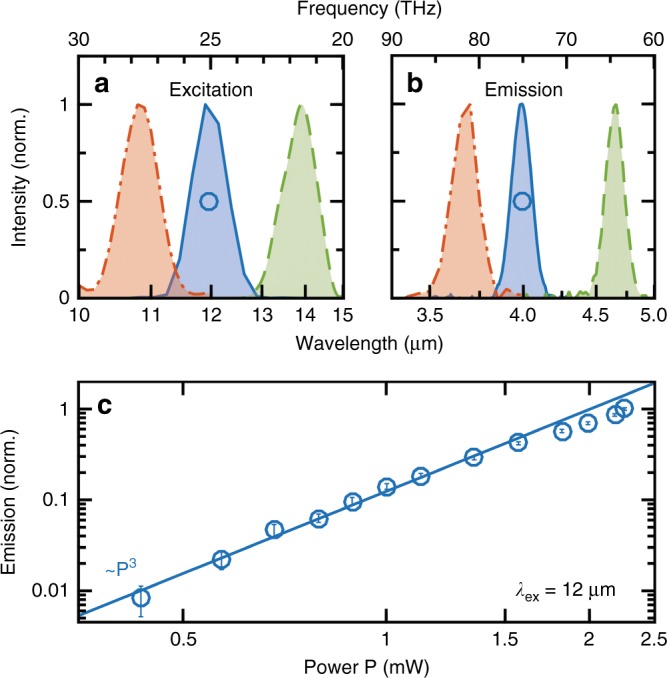
Third harmonic emission spectra and nonlinear power dependence. **a** The excitation spectra and **b** the corresponding third harmonic emission spectra from single resonant double-rod antennas at excitation wavelengths of 10.7, 12, and 14 µm (red, blue and green, respectively). **c** The nonlinear power dependence of the THG emission on a double-logarithmic scale for a 12 µm excitation wavelength (circles). The line of cubic proportionality proves the third harmonic character of the radiation and shows the start of saturating behavior for high excitation powers beyond 1.5 mW

Figure 3a and b shows the emission spectrum under three different excitation conditions. The power dependence of the emitted THG radiation from the antenna center (Fig. 3c) with a cubic power exponent further proves that we indeed observe third harmonic emission in this experiment. At high pump intensity levels, the curve demonstrates that the efficiency of the nonlinear emission starts to deviate from a third-order power law. This could be caused by transient heating of the antenna that modifies the dielectric behavior of Ge or by charge carrier excitations that slightly increase the reflection losses.

Taking into account the losses of the optical elements employed for the collection of the third harmonic emission, we estimate a yield of the nonlinear frequency conversion (defined as the number of third harmonic photons generated per excitation photon) that is higher than 10^−6^ for a single antenna emitter illuminated by 25 nJ driving pulses. This calculation does not include the limited collection aperture of the condenser objective with respect to the emission pattern of the THG. If a standard dipole emitter coupled to the antenna gap is considered to simulate the third harmonic radiation pattern, the total energy conversion ratio can be estimated to be ~10^−5^ with a THG energy on the order of 1 pJ per pulse.

**Fig. 4:**
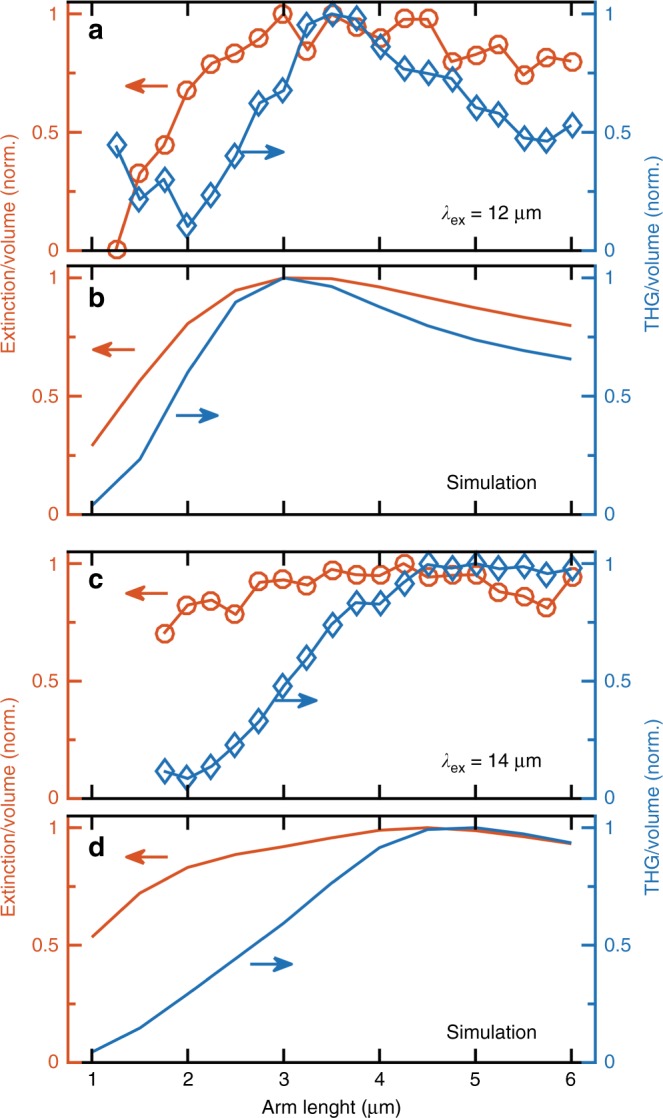
Measurement and simulation of the extinction and THG emission for antennas of various arm lengths. **a**, **c** The normalized extinction (red circles) and third harmonic emission (blue diamonds) per unit antenna volume measured for double-rod antennas of various lengths at a gap width of 300 nm excited at 12 and 14 µm central wavelengths, respectively. **b**, **d** Corresponding FDTD simulation results for both excitation conditions

Finally, we investigate the dependence of both the linear scattering cross section and the third harmonic emission on the antenna arm length. The extinction and emission data are extracted from confocal microscopy raster scans similar to those in Fig. 2 for a set of different antennas (a set of images is presented in Fig. S4 of the supplementary information). The arm length is varied between 1 and 6 µm at a constant gap width of 300 nm. Figure 4a demonstrates the measurement results for an excitation wavelength of 12 µm. By normalizing over the antenna volume, a resonance for an arm length of ~3.5 µm becomes visible. As expected from the nonlinear scaling of the excitation, this effect becomes more prominent in THG emission. For antennas with an arm length <1.5 µm, no significant third harmonic emission is detected, whereas antennas display a broad peak of efficiency when the arm length is fixed between 3 and 5 µm. Above these values, the nonlinear emission becomes less efficient because of the off-resonance conditions. The measurement is repeated in Fig. 4c for an excitation wavelength of 14 µm displaying a shift of the resonance to longer antennas with an arm length of ~5 µm.

Figure 4b and d displays the results of three-dimensional finite-difference time-domain (FDTD) simulations that reproduce the observed behavior with high fidelity, taking the numerical aperture of the two objectives into account. The local THG is evaluated by considering the third power of the field intensity integrated over the whole antenna volume. Moreover, the efficiency of the THG radiation process is also included by considering the coupling between a dipole emitter at the third harmonic wavelength and the antenna and by projecting the resulting field distribution to the far field. In this way, both the localized plasmon resonances at the fundamental frequency (which play by far the major role by virtue of the third-order nonlinearity of the process) and any possible photonic mode at the third harmonic frequency (which lies above the plasma frequency) are taken into account. The plasmonic antenna resonance involved in the enhancement of the THG efficiency is also demonstrated by simulations of the absorption spectra and of the local field distributions for all the investigated antennas, as displayed in Figs. S1 and S2 of the supplementary information. In the Ge-on-Si plasmonic platform, the high index of the substrate results in two distinct resonances: one with hot spots located mainly towards the Si substrate and one with hot spots located mainly towards the air^15^. We exploit the latter because (i) it exists at a higher frequency for a given doping level, therefore allowing for easier coverage of the whole mid-IR range, and (ii) the positions of the hot spots are more favorable in view of local interactions with molecules and emitters in general.

## Discussion

In conclusion, we were able to demonstrate for the first time coherent nonlinear emission from plasmonic subwavelength structures in the mid-IR frequency range. This achievement is enabled by the crucial advancements in the growth of epitaxial group-IV semiconductors and of heavily doped germanium on silicon substrates in particular. Our results pave the way for new methods in mid-IR near-field microscopy employing germanium nanostructures^29^ and for the sensing of molecules based on their vibrational absorption fingerprints. This capability combined with integration in a CMOS platform will allow targeting sensitive applications in biomedicine for the observation of health markers, international security for the detection of hazardous compounds and even environmental protection, for example, by monitoring the emissions of combustion engines. In addition, semiconductor plasmonics are promising for fundamental studies of nonlinear light-matter interactions. In perspective, new insight into controversial questions about the origin of the nonlinear susceptibility in nanostructures^23,28^ and its spectral dispersion can be gained by tuning the frequency of the plasma edge in doped germanium, thus providing a unique degree of freedom that is inaccessible with standard metals. Finally, combined with the all-optical ultrafast generation of free carriers in intrinsic Ge nanostructures^24^, this work opens up the possibility for active control of coherent mid-infrared light sources with unprecedented spatiotemporal confinement.

## Materials and methods

### Heavily doped Ge film growth and material characterization

As a first fabrication step, a highly n-doped Ge epilayer was grown on a high-resistivity Si(001) substrate by low-energy plasma-enhanced chemical vapor deposition (LEPECVD)^30^. The deposition was performed at 500 °C with a growth rate of ~1 nm/s. In situ n-type doping was achieved by adding 0.035 standard cubic centimeter per minute (sccm) of PH_3_ to 20 sccm of GeH_4_, yielding an active doping of 2.5 × 10^19^/cm^3^, which corresponds to a plasma frequency of ~31 THz (9.7 μm wavelength). The thin film reflectivity spectrum is recorded using Fourier transform infrared spectroscopy and allows extraction of the dielectric function of the material (Fig. 1a, b) by a combination of Drude-like modeling and the use of the Kramers–Kronig relations^16^ (see also Supplementary information). It should be noted here that recent works also demonstrated that the same growth technique, when combined with postgrowth annealing procedures, allows plasma frequencies up to ~95 THz (3.1 µm wavelength) to be reached^31,32^.

### Antenna fabrication

From these heavily doped Ge films, isolated antenna structures (Fig. 1c) are fabricated via electron-beam lithography with hydrogen silsesquioxane resist and anisotropic reactive ion etching with fluorine chemistry^33^. The half-wavelength length of the two arms in the double-rod antenna geometry is selected to maximize the resonantly enhanced currents. Considering the refractive index of the material and the environment, numerical simulations predict that the first-order localized plasmon-polariton resonance occurs for an arm length of 3 to 4 µm when excited at a wavelength of ~12 µm with linearly polarized light. The use of state-of-the-art nanofabrication technology enables relatively large structures to be fabricated close to perfection with steep sidewalls, sharp edges and reproducibility to within a nm of the design^33^.

### Mid-IR laser microscopy setup and antenna characterization

To excite the resonant antenna structures, we developed a high-power femtosecond laser system^34^ based on a regenerative Yb:KGW amplifier with a repetition rate of 50 kHz. The fundamental pulses at a wavelength of 1028 nm with a duration of 250 fs drive a noncollinear optical parametric amplifier (NOPA)^35^ that generates broadband pulses tunable between 1050 and 1400 nm. Intense mid-IR pulses are subsequently produced via nonlinear phase-matched difference frequency mixing of the NOPA pulses (pulse energy up to 2.2 µJ) with 36-µJ pulses from the Yb:KGW amplifier in a GaSe crystal^36^. Depending on the crystal thickness, phase matching angle and NOPA wavelength, either broadband mid-IR transients spanning from 8 to 20 µm in wavelength or narrowband intense pulses of up to 100 nJ tunable over the same spectral range can be generated. For these experiments, the driving mid-IR pulses are set to a duration of 300 fs at a bandwidth of 1.5 THz.

A custom-built confocal microscope consisting of two dispersion-free gold-coated reflective Cassegrain objectives with a numerical aperture of 0.5 and working in a transmission geometry is used to study the linear and nonlinear mid-IR response of individual antenna structures. The first objective focusses the driving pulses onto the antenna samples, yielding excitation fields of up to 5 MV/cm and reaching a diffraction-limited spot size of approximately one wavelength. The second objective collects the transmitted radiation as well as the nonlinear emission. A pinhole with a diameter of 150 µm in the image plane reduces the detection field of view to enhance the lateral resolution, rejects stray light coming from outside the focal volume and lowers the depth of field to better discriminate between the substrate bulk emission background and the antenna signal. The detection of the transmitted and emitted nonlinear radiation is performed via electro-optic sampling (EOS) in 80-µm-thick GaSe^36^ or with a liquid-nitrogen-cooled mercury cadmium telluride (MCT) detector with low-noise, lock-in readout. To discriminate between the fundamental and third harmonic radiation, crystalline filters of InSb (a long wavelength pass filter with a transmission edge at 7 µm wavelength), sapphire (a 5 µm short wavelength pass filter), and CaF_2_ (a 9 µm short wavelength pass filter) are employed. A monochromator with blazed mid-IR gratings was employed to record the exact emission spectrum from the samples.

### Simulation of the antenna response

Three-dimensional finite-difference time-domain simulations (FDTD solutions, Lumerical Inc.) were performed to reproduce the observed extinction (see Fig. S1 in the supplementary information) and emission spectra. The dielectric constants of the heavily doped Ge material and of the underlying Si substrate were experimentally characterized as described above and used for the simulations. The antenna arms and the gap are defined by a rectangular mesh with a cell size of 15 nm, while the remaining simulation volume (vacuum and Si substrate) is covered by a high-quality conformal mesh generated by the software (see Fig. S2 in the supplementary information). The antennas are illuminated with a Gaussian source at the fundamental wavelength, featuring the same numerical aperture of 0.5 as the illumination objective in the experimental setup. Convergence is accurately checked by terminating the calculations only when the total energy inside the simulation volume is reduced to a fraction of 10^−5^ of the originally injected energy. The influence of the numerical aperture of the collection objective in the extinction maps was taken into account as well by considering the radiation pattern of the antennas at the fundamental wavelength (far-field projection of the emission of a dipole coupled to the antenna gap). To model the emission of the nonlinear signal and reproduce its dependence on the arm length, we calculate the third power of the electric field intensity integrated over the whole antenna volume, mimicking a standard third-order bulk nonlinear process inside the antennas. The third harmonic angular emission pattern of the antenna was simulated considering a dipole emitter, peaking at the third harmonic wavelength, coupled to the antenna gap as a simplistic emission model that allowed us to evaluate the fraction of THG falling inside the numerical aperture of the collection objective. This simple approach already reproduces the experimental data with high fidelity.

## Supplementary information

Supplemental Material
