# Supplementary material for: Plasmonic mid-infrared third harmonic generation in germanium nanoantennas
Source: Light Sci Appl. 2018 Dec 12;7:106. doi: 10.1038/s41377-018-0108-8 (PMC6290006; doi:10.1038/s41377-018-0108-8)
Supplement: Supplementary file 1 — Supplemental Material [file 41377_2018_108_MOESM1_ESM.docx]

**Supplementary Information for:**

**Plasmonic Mid-infrared Third Harmonic Generation in Germanium Nanoantennas**Marco P. Fischer^1^, Aaron Riede^1^, Kevin Gallacher^2^, Jacopo Frigerio^3^, Giovanni Pellegrini^4^,
Michele Ortolani^5^, Douglas J. Paul^2^, Giovanni Isella^3^, Alfred Leitenstorfer^1^, Paolo Biagioni^4^,
and Daniele Brida^1,6,*^

^1^ Department of Physics and Center for Applied Photonics, University of Konstanz, D-78457 Konstanz, Germany
^2^ School of Engineering, University of Glasgow, Rankine Building, Oakfield Avenue, Glasgow, G12 8LT, UK
^3^ L-NESS, Dipartimento di Fisica del Politecnico di Milano, Via Anzani 42, 22100 Como, Italy
^4^ Dipartimento di Fisica, Politecnico di Milano, Piazza Leonardo da Vinci 32, 20133 Milano, Italy
^5^ Department of Physics, Sapienza University of Rome, 00185 Rome, Italy
^6^ Physics and Materials Science Research Unit, University of Luxembourg,162a avenue de la Faïencerie, L-1511 Luxembourg, Luxembourg
^*^ e-mail: [daniele.brida@uni.lu](mailto:daniele.brida@uni-konstanz.de)


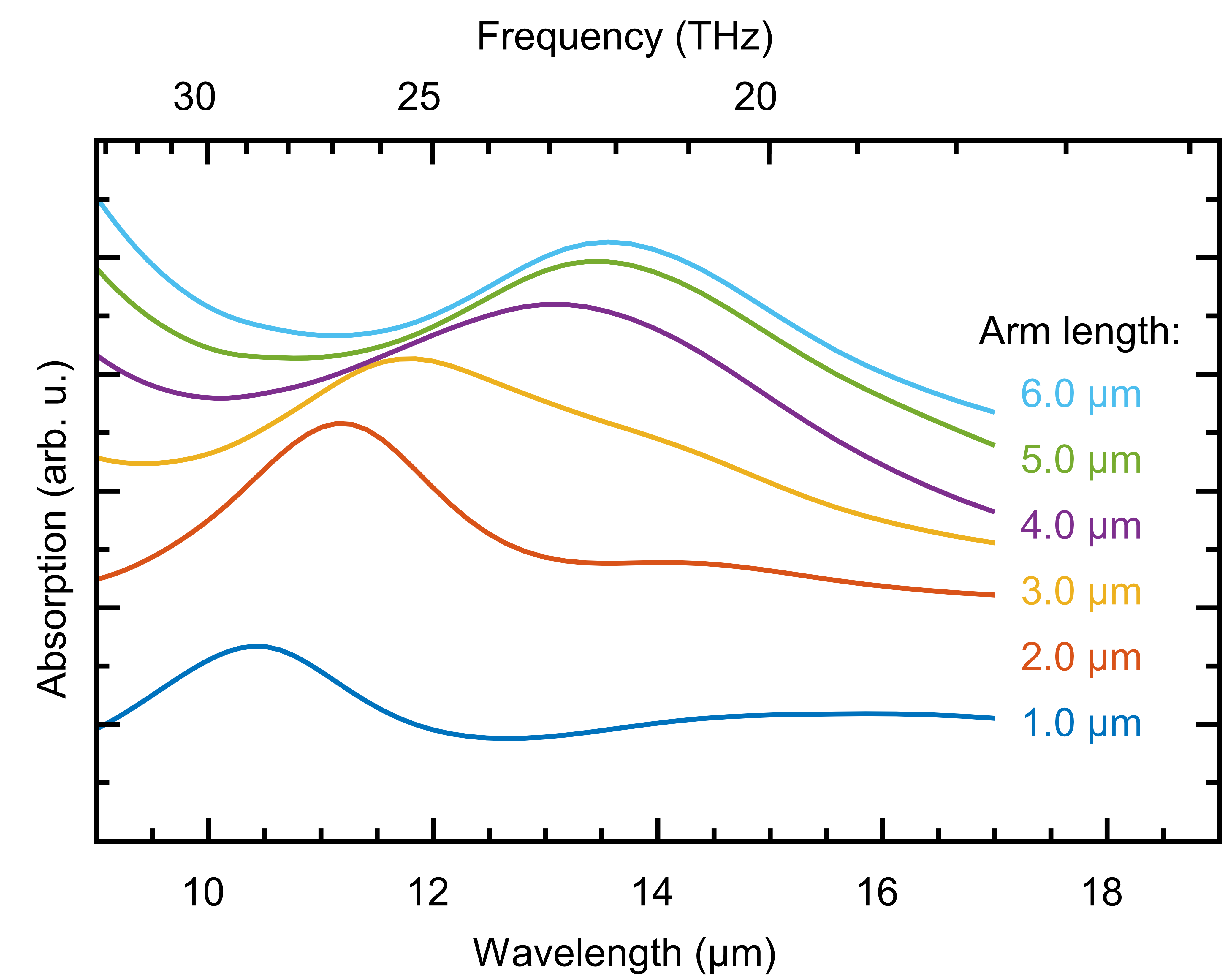


**Figure S1 | FDTD simulation of absorption spectra of heavily-doped Ge double rod antennas.** Simulated absorption spectra for six heavily doped Ge antennas on silicon substrates with arm lengths between 1 and 6 µm. For this simulation the confocal microscope geometry and apertures are taken into account. For increasing arm lengths, the plasmonic resonance shifts from 10.5 µm to about 14 µm wavelength, which is below the plasma edge wavelength of 9.5 µm of the heavily doped germanium.


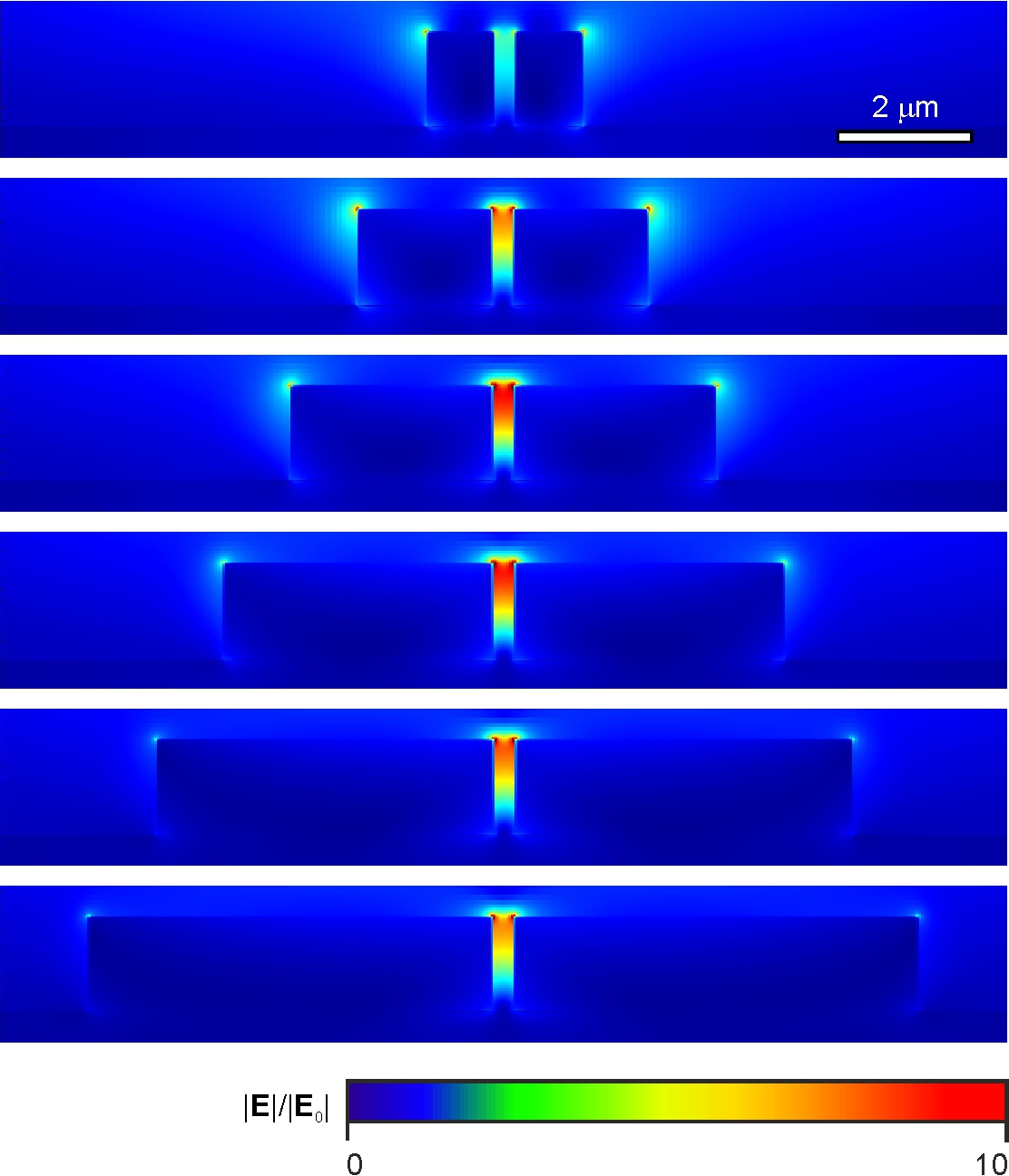


**Figure S2 | FDTD simulations of the antenna near field distribution.** Simulated near-field distribution of six heavily-doped Ge double rod antennas on silicon substrates with arm lengths between 1 and 6 µm. The respective field enhancement is displayed according to the color scale valid for all panels. The antennas are depicted in side-view on top of the substrate. Gaussian illumination is modelled for pulses at a central wavelength of 12 µm taking the illumination objective aperture into account. At this wavelength only the plasmonic mode located at the top interface of the antennas is excited (Ref. 16 of the main text). In the gap region a maximum field enhancement factor of about 10 can be observed.


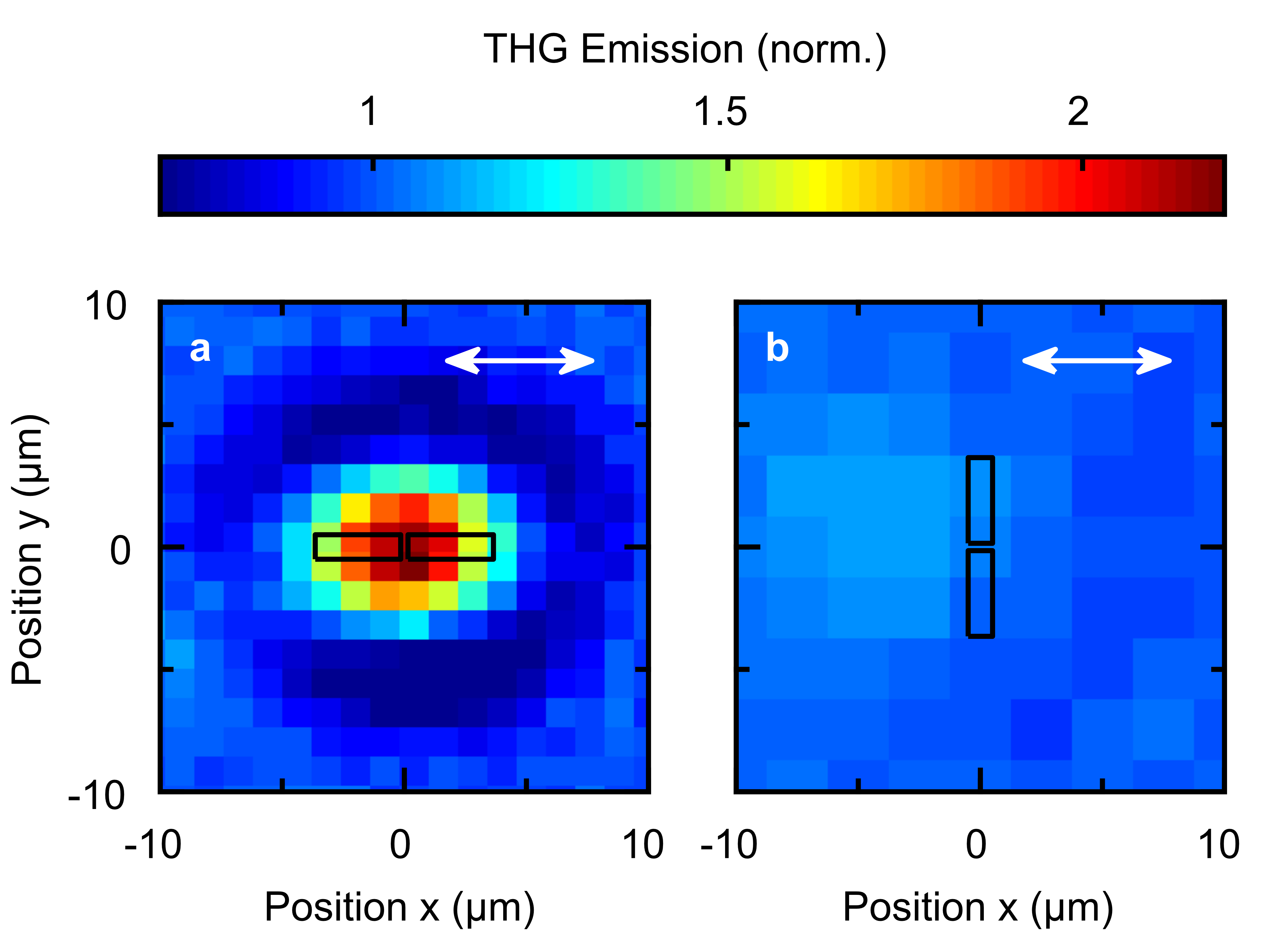


**Figure S3 | Polarization dependence of the third harmonic emission of a Ge double rod antenna.** **a**, Illumination with field polarization parallel to the antenna axis. **b**, Illumination with the field polarization perpendicular to the antenna axis. In both panels, the arrows indicate the polarization direction of the incident excitation beam.


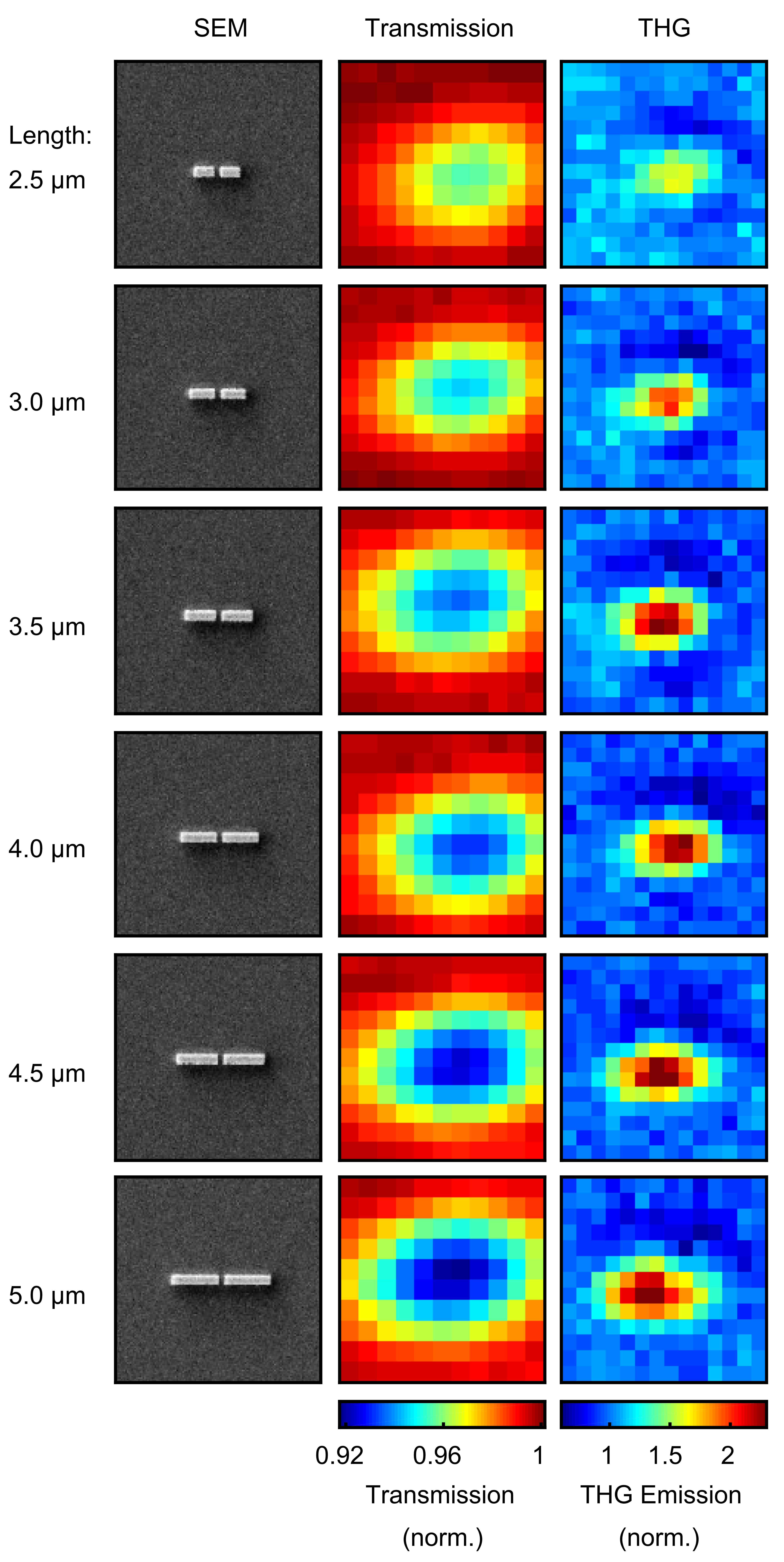


**Figure S4 | Linear and nonlinear confocal microscope images of a set of isolated antenna structures with increasing arm length.** **Left column**, Scanning electron micrographs of several double rod antennas with increasing arm. **center**, Spatially resolved transmission maps of these antennas at 12 µm illumination (common colour bar at the bottom). **right**, Nonlinear emission maps at the same excitation wavelength. The third harmonic generation (THG) wavelength is 4 µm. The common colour bar at the bottom is normalised to the substrate background emission. Each panel shows an area of 20 x 20 µm².


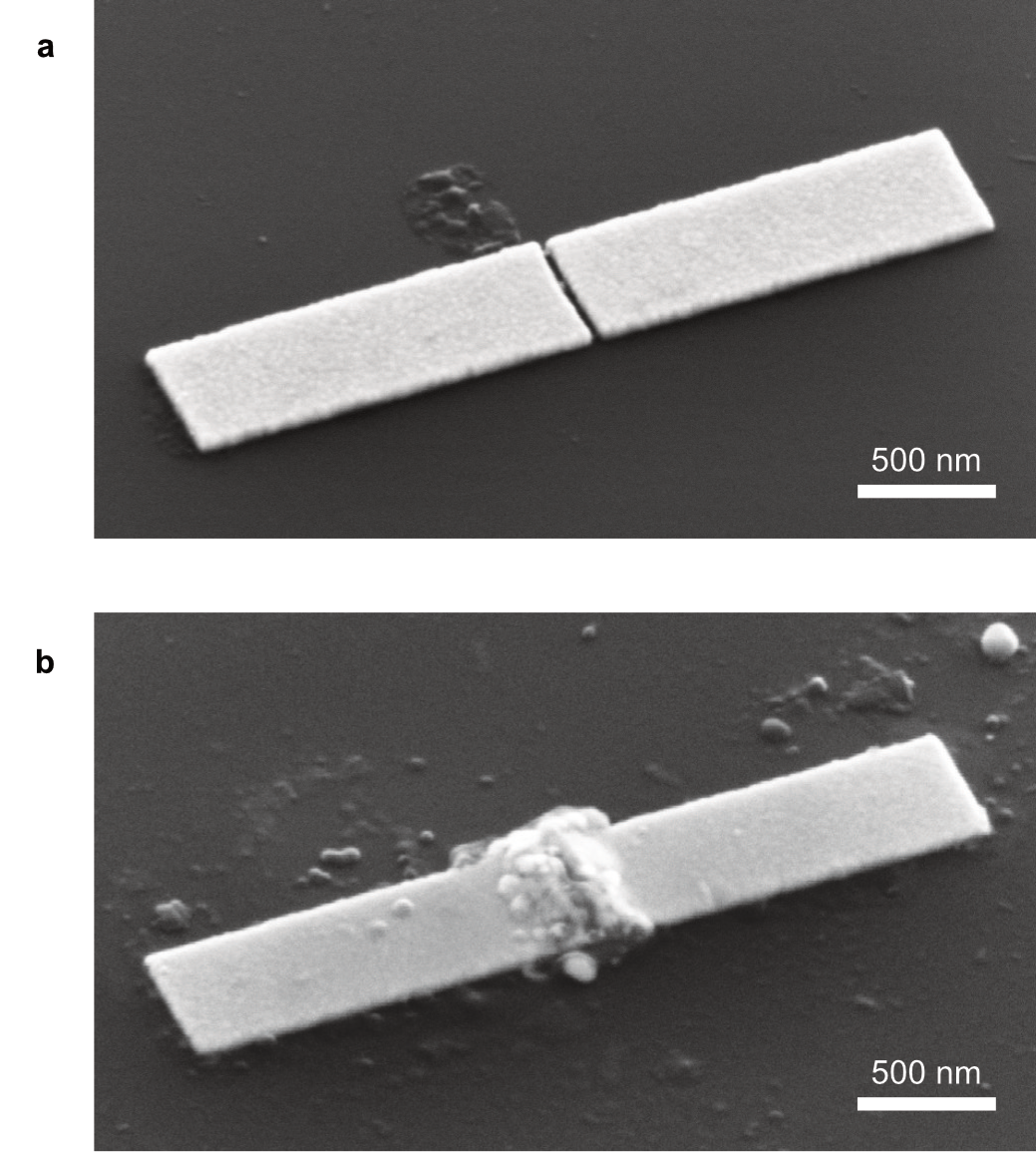


**Figure S5 | Gold double rod antenna structures on silicon substrate. a**, SEM micrograph of a gold antenna designed to be resonant at 11 µm. **b**, Same antenna after prolonged illumination with pulses centered at a wavelength of 11 µm. The gap shows strong electro-migration effects while no third harmonic emission could be detected.

**
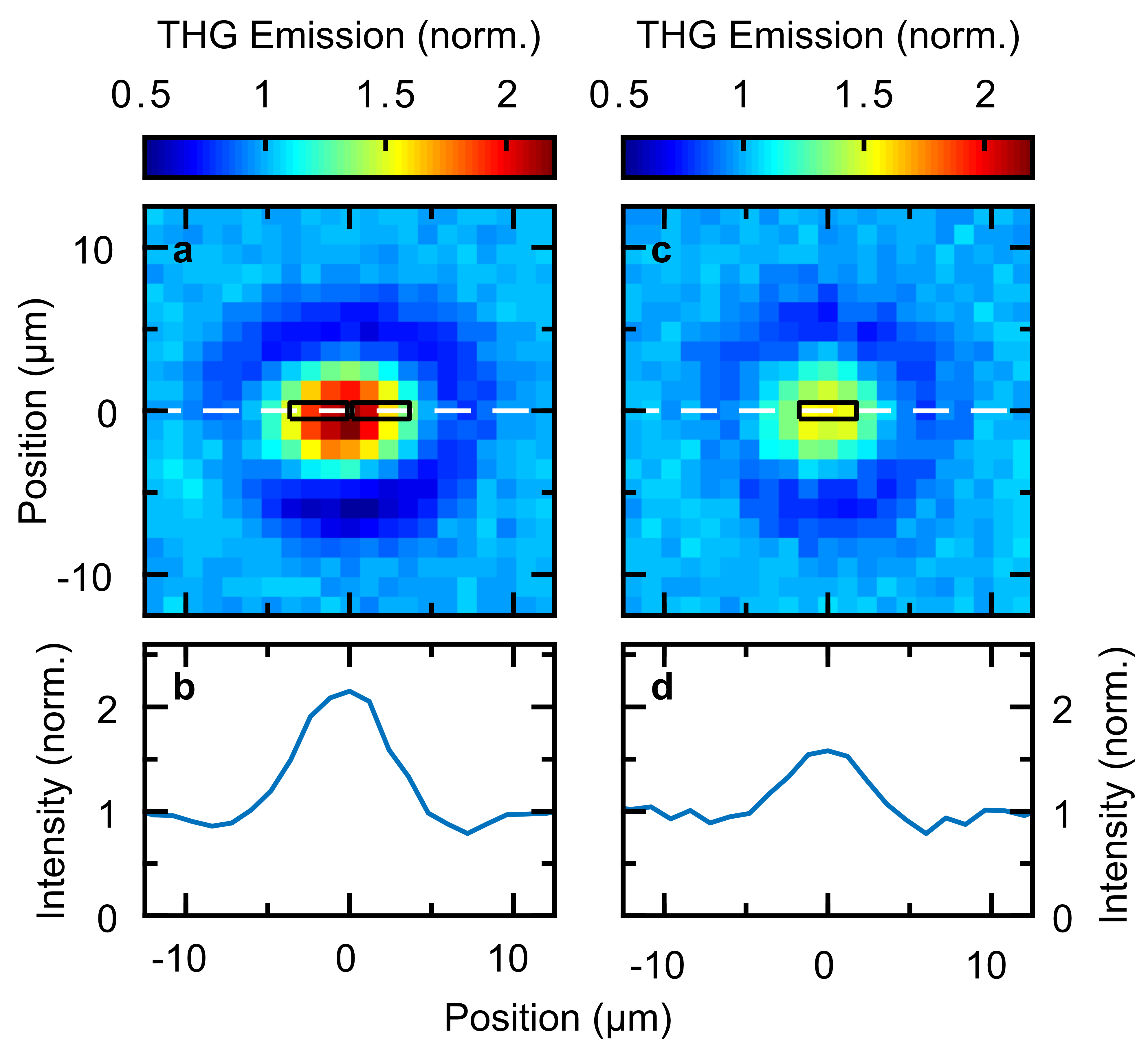
**

**Figure S6 | Comparison of the third harmonic emission of a Ge double rod antenna and a Ge single rod antenna.** **a**, Spatially resolved third harmonic emission intensity from a double rod antenna with arm length 3.5 µm normalized to the substrate background emission. **b**, Cut of the emission map through the center of the antenna (along the white dashed line). **c,** Spatially resolved third harmonic emission of a single rod antenna with arm length 3.5 µm. **d**, Cut of the emission map.

**Dielectric function determination**

The dielectric function shown in Figure 1a of the main text has been determined from the experimental normal incidence reflectance of the as-grown epitaxial germanium film by inverting the following Fresnel relations:

$$R(\omega)\cong\frac{r_{12}+r_{23}e^{4\pi in(\omega)d}e^{-\alpha(\omega)d}}{r_{12}+{r_{12}r}_{23}e^{4\pi in(\omega)d}e^{-\alpha(\omega)d}}$$

where $r_{jk}$are the frequency (ω) dependent complex Fresnel reflection coefficients of each interface. Subscripts 1, 2 and 3 refer to vacuum, germanium film and silicon substrate respectively. $n(\omega)$ and $\alpha\left( \omega\right)$ are the germanium refractive index and absorption coefficients and *d* is the film thickness. Other details can be found in Ref. 16 of the main text.
